# Acupuncture for Relief of Gag Reflex in Patients Undergoing Transoesophageal Echocardiography—A Protocol for a Randomized Placebo-Controlled Trial

**DOI:** 10.3390/medicines7040017

**Published:** 2020-03-31

**Authors:** Taras I. Usichenko, Irina Müller-Kozarez, Stephan Knigge, Raila Busch, Mathias Busch

**Affiliations:** 1 Department of Anesthesiology, University Medicine of Greifswald, 17475 Greifswald, Germany; stephan.knigge@med.uni-greifswald.de; 2 Department of Anesthesia, McMaster University, Hamilton, ON L8S 4K1, Canada; 3 Department of Internal Medicine, University Medicine of Greifswald, 17475 Greifswald, Germany; irina.mueller-kozarez@uni-greifswald.de (I.M.-K.); raila.busch@uni-greifswald.de (R.B.); Mathias.Busch@med.uni-greifswald.de (M.B.); 4 German Center for Cardiovascular Research (DZHK), 17475 Greifswald, Germany

**Keywords:** transesophageal echocardiography, gag reflex, acupuncture

## Abstract

**Background:** Gagging during transesophageal echocardiography examination (TEE) can be distressing and even dangerous for patients. The needling of acupuncture point CV24 was described to be effective in reducing the gag reflex during TEE in patients with ischemic stroke or transient ischemic attack. **Methods:** We describe a proposal for a prospective, randomized, patient, practitioner and assessor-blinded, single-center trial with two arms/groups; real acupuncture will be compared to placebo acupuncture. A total of 60 (30 per group) patients scheduled for elective TEE in order to exclude a cardiac embolic source, endocarditis or for valve failure evaluation will be recruited according to patients’ selection criteria and receive either indwelling fixed intradermal needles at acupoints CV24 and bilateral PC6 or placebo needles at the same areas. Patients, the practitioners who will perform the TEE procedure, and the assessor of the outcome measures will be unaware of the group’s (real or placebo) allocation. **Results:** The primary outcome is the intensity of gagging, measured using verbal rating scale (VRS-11) from 0 = no gagging to 10 = intolerable gagging. Secondary outcomes include the incidence of gagging, the use of rescue medication, patients’ satisfaction with relief of unwanted side effects during TEE procedure, success of patients’ blinding (patients’ opinion to group allocation), heart rate and oxygen saturation measured by pulse oxymetry. **Conclusions:** To study the effects of acupuncture against gagging during TEE, we test the needling of acupoints CV24 and PC6 bilaterally. A placebo acupuncture is used for the control group. Trial registration number: NCT NCT0382142.

## 1. Introduction

The gag reflex is a part of a normal defense mechanism, intended to prevent unwanted, irritating, or toxic material or fluids from entering the trachea, pharynx or larynx. It is a polysynaptic reflex, which arises at the cortical level and is composed of a palatal and a pharyngeal response. Palatal response comprises an upward movement of the soft palate with an ipsilateral deviation of the uvula, whereas the pharyngeal response means the contraction of the pharyngeal wall [1,2]. Concomitant features include salivation, eye watering, coughing, sweating, head withdrawal, fainting or even a panic attack [3]. The gag reflex can be stimulated by touching trigger areas like the posterior pharyngeal wall, tonsillar area, and the base of the tongue, palate and uvula.

With the growing number of diagnostic and therapeutic procedures where manipulation in the pharyngeal area is necessary (e.g., with fiberoptic probes), gagging became a common clinical problem, with an incidence of more than 40% despite use of local anesthesia and sedation [4,5]. Gagging makes diagnostic and therapeutic procedures distressing and often difficult or even impossible to perform. Various interventions are used to control the gag reflex, for example, antiemetic medication, sedatives, local and general anesthetics, herbal remedies, behavioral therapies, acupressure, acupuncture, and prosthetic devices; however, there is no evidence favoring any specific treatment of the gag reflex in clinical conditions [5].

**Figure 1:**
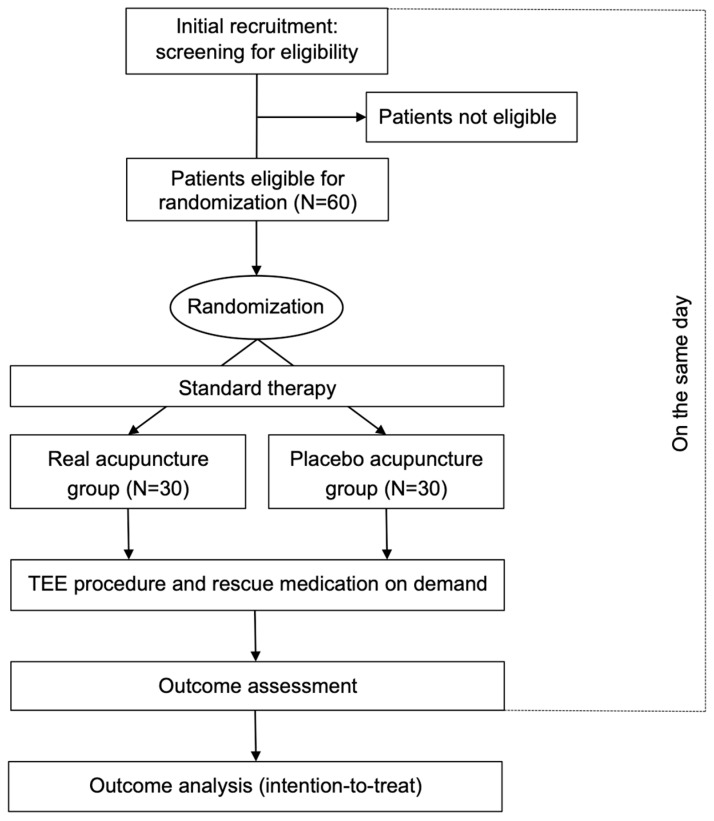
Flow of the investigation. TEE: transoesophageal echocardiography.

The needling of acupuncture points CV24 and PC6 (Figure 1), is suggested as a complementary or even alternative option for the treatment of gagging and concomitant nausea during various medical procedures [6,7,8,9,10,11].

Acupuncture of CV24 and PC6 is commonly used to alleviate the gag reflex in dentistry in adult and in pediatric patients. However, the effectiveness of acupuncture for this condition was suggested on the basis of several pilot case series or non-robust experimental investigations [6,7,10,11]. Beyond dentistry, the needle stimulation of acupoint CV24 was suggested to be effective for suppressing the gag reflex in patients with ischemic stroke or transient ischemic attack undergoing transesophageal echocardiography (TEE) [9]. In this pilot investigation, acupuncture was superior to a sham procedure, was easy to apply and effective in reducing the gag reflex during TEE.

We are going to evaluate whether adding acupuncture to current clinical standards in reducing gag reflex in patients undergoing diagnostic TEE is a secure, effective and easy-to-use method. For this purpose, we will compare the acupuncture of CV24 and PC6 with a placebo procedure in a two-arm randomized controlled investigation, where the patients, cardiologists and outcome assessors will be blinded to group allocation.

## 2. Materials and Methods

### 2.1. Study Design

This investigation is a prospective randomized, patient, practitioner and assessor-blinded, single-center trial at the University Medicine of Greifswald with two arms/groups: real acupuncture will be compared to placebo acupuncture. The total duration of the investigation for each patient is approximately 30 min. A total of 60 (30 per group) patients scheduled for elective transesophageal echocardiography (TEE) will be recruited according to patients’ selection criteria. TEE is performed in preparation for an electrophysiological procedure, to exclude a cardiac embolic source or endocarditis or for valve failure evaluation. Patients, the practitioners who perform the TEE procedure and the assessor of the outcome measures, are unaware (“blinded”) regarding the group allocation. Only the acupuncturist, who performs the randomization, will know the group allocation. The local ethics committee approved the protocol of the investigation. The schedule of enrolment, interventions and assessment is given in the Supplementary Figure S1 according to SPIRIT recommendations

### 2.2. Eligibility Criteria

Patients between 19 and 65 years old with an American Society of Anesthesiologists physical status of I to III, scheduled for elective ambulatory transesophageal echocardiography (TEE) without sedation, without previous opioid and psychotropic medication, who were able to use the verbal rating scale 0–10 (VRS-11) were recruited in this investigation. Patients who were unable to give the written informed consent or had a skin infection at the sites of acupuncture or reported a history of psychiatric disease, radio- or chemotherapy or peripheral polyneuropathy were not included. Patients with a congenital absence of gag reflex and pregnant women were not included in this investigation. The data of patients where the usual TEE schedule had to be changed or who developed severe complications during the investigation were excluded from the final analysis.

### 2.3. Group Allocation and Randomization

During the standard pre-procedural examination, the patients, screened according to eligibility criteria (s. above), will be informed about the possibility to receive either real acupuncture (RA) or placebo acupuncture (PA) by chance in addition to standard therapy for relief of the gag reflex during TEE and will be asked if they would like to take part in the investigation. If they agree, they will have to sign their informed consent and will be randomly allocated to RA (30 patients) or PA (30 patients) prior to the TEE procedure (Figure 1).

The acupuncturist will perform the allocation procedure using the randomly-generated-by-computer list of numbers 1 and 2: the patients who will receive RA will be assigned to one of two numbers; one number represents the RA group and the other represents placebo. The randomization is concealed from patients and practitioners; only the acupuncturist will be aware of the group allocation.

### 2.4. Current Standard Treatment for TEE Procedure

According to our hospital standard operating procedure for TEE preparation, all patients will receive three puffs (approximately 30 mg) of 10 % lidocaine pump spray (Astra Zeneca) prior to acupuncture/placebo acupuncture and TEE procedure. In case the gag intensity reaches 10 points on VRS-11 and the insertion of TEE probe is impossible, midazolam 1–2 mg will be administered intravenously. If the maximal dose of 5 mg midazolam is reached and the patient still experiences gagging, which precludes the insertion of the TEE probe, than incremental doses of 10 mg propofol as a rescue medication will be allowed until the TEE probe is inserted.

### 2.5. Study Procedure

**Figure 2:**
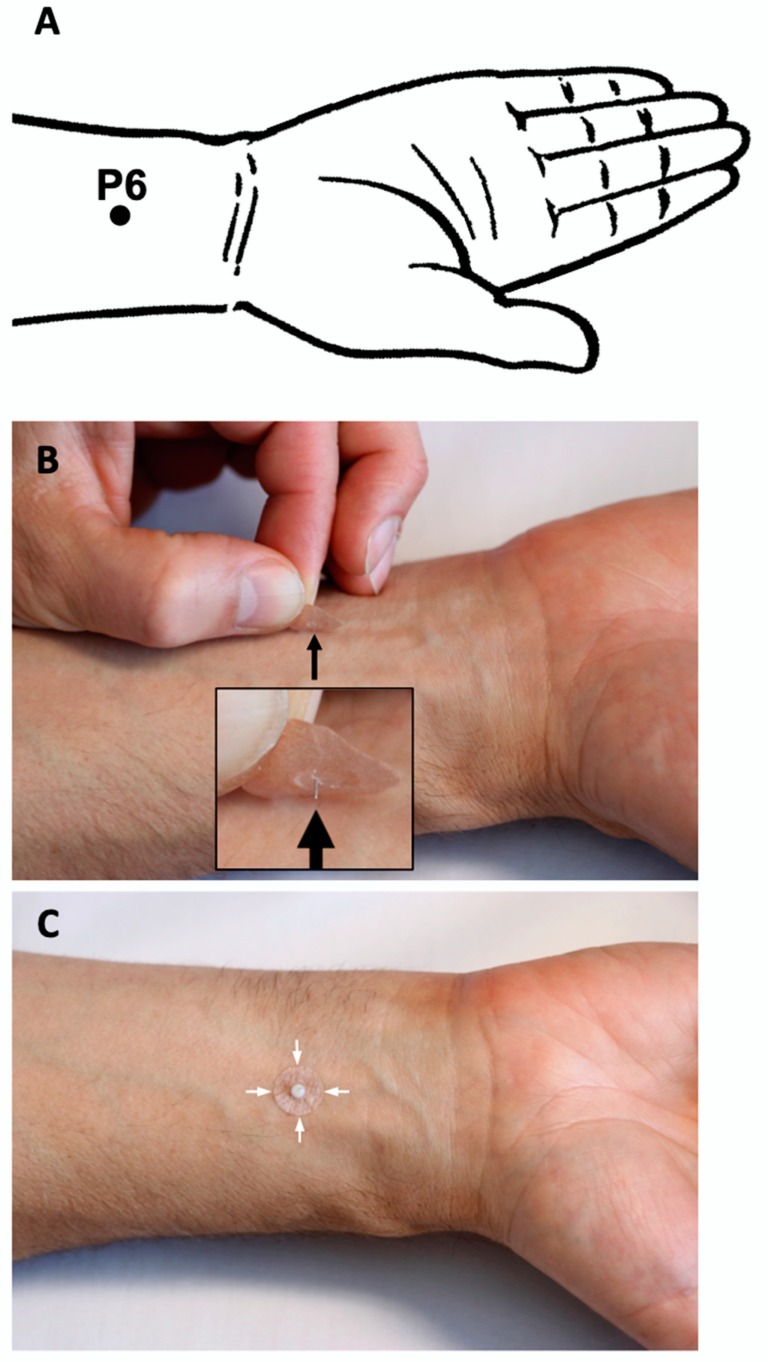
(**A**) Acupoint *Neuguan* PC6 is situated on the medial side of the lower arm between the tendons of M. flexor carpi radialis and M. palmaris longus 3–4 cm over the wrist crease; (**B**) disposable indwelling acupuncture New Pyonex needle (black arrow) with a diameter 0.2 and length 1.5 mm, embedded in skin-colored adhesive tape, over the acupoint PC6. (**C**) New Pyonex needle in situ. White plastic hemisphere is seen over the round adhesive tape (white arrows).

In the RA group, the acupuncturist will apply the needles to *Chengjiang* CV24 and both *Neiguan* PC6 (bilaterally) acupuncture points prior to the TEE procedure. Acupoint PC6 is situated on the medial side of the lower arm between the tendons of M. flexor carpi radialis and M. palmaris longus 3–4 cm over the wrist crease (Figure 2).

**Figure 3:**
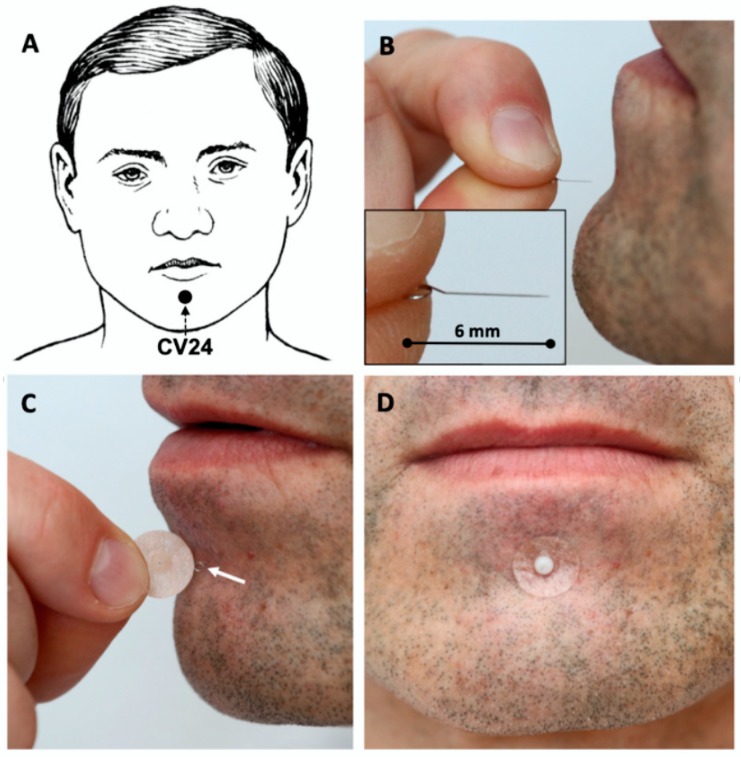
(**A**) acupoint *Chengjiang* CV24 is situated on the ventral midline in a mento-labial groove. (**B**) disposable intradermal “Spinex” needles with diameter 0.14 and length 6 mm over the CV24 acupoint. (**C**) the loop of the inserted “Spinex” needle is seen on the surface of the skin (white arrow), New Pyonex Placebo adhesive tape in the fingers. (**D**) New Pyonex Placebo adhesive tape is attached over “Spinex” needle.

Acupoint CV24 is situated on the ventral midline in a mento-labial groove (Figure 3).

The choice of acupuncture points is based on the findings from previous investigations on the treatment of gagging in patients during medical procedures [6,7,8,9,10,11] and was performed according to Standard for Reporting Interventions in Clinical Trials of Acupuncture (STRICTA) recommendations (Table 1) [12].

For the acupuncture of PC6 points, disposable indwelling acupuncture New Pyonex needles (diameter 0.2 and length 1.5 mm, manufactured by Seirin Corp., Shizuoka, Japan) embedded in skin-colored adhesive tape will be used (Figure 2B,C). Acupuncture of point CV24 will be done using disposable intradermal “Spinex” needles (diameter 0.14 mm and length 6 mm; Seirin Corp., Shizuoka, Japan, Figure 3B). the Spinex needle will be inserted and its loop, which remains on the surface of the skin (Figure 3C), will be attached using New Pyonex Placebo needles (Figure 3D). A New Pyonex Placebo needle, which was designed for a placebo control procedure in auricular acupuncture studies, has the same appearance as the New Pyonex needle but consists of self-adhesive tape only, without the needle itself (Figure 3C).

In patients who are assigned to the PA group, the acupuncturist will apply the New Pyonex placebo needles to the same areas where the needles for RA would be inserted. In order to provide the feeling of “pricking” during the application of New Pyonex placebo needles, the skin areas around acupuncture points will be examined using the using SVESA neural pen (Neuralstift SVESA 1070, SVESA, Muenchen, Germany). The SVESA neural pen is commonly used in acupuncture practice for the identification of skin areas with lower skin resistance, which are attributed to acupuncture points, as was described elsewhere [13]. The neural pen has a thin tip, which produces the feeling of needle insertion, if certain pressure is applied to the skin. Acupuncturist will tell the patients from PA group that he is using the neural pen “to find” the acupuncture points, however he will use it to produce a pressure with the tip of neural pen (resembling the pricking of the needle) and attach New Pyonex placebo needles at the areas of the skin near the acupuncture points, but not at the acupoints exactly.

The acupuncture needles or placebo needles will be placed immediately before the TEE procedure, retained in situ during the diagnostic procedure, and will be taken out after the TEE probe is withdrawn. Before the needle’s insertion, the skin will be completely disinfected with alcohol swabs.

### 2.6. Outcome Measures

The intensity of gagging, reported by the patient using the verbal rating scale (VRS-11) from 0 = no gagging to 10 = maximally uncomfortable under gagging, was chosen as a primary outcome measure (Supplementary Figure S1). The incidence of gagging, nausea and vomiting, reported by the patient, will be recorded as secondary outcomes. Patients will also be asked about the intensity of nausea, using VRS-11 (from 0 = no nausea at all to 10 = maximal uncomfortable nausea). The practitioner who performs the TEE procedure will assess the intensity of gagging using VRS-11 (from 0 = no gagging at all to 10 = maximal gagging), record the requirement of midazolam and propofol as a rescue medication, and record the heart rate and oxygen saturation and potential side effects of acupuncture. Patients’ satisfaction with relief of unwanted side effects during TEE procedure will be evaluated using VRS-5 (from 1 = excellent to 5 = unsatisfactory); both patients and practitioners (TEE examiner) will be interviewed regarding their opinion of group allocation.

### 2.7. Sample Size

In order to calculate the appropriate sample size, we set the level of significance to 0.05 and power at 80%. Based on the results of investigation published by Rösler et al. [9] and expecting to find a 30% difference in gagging intensity between the acupuncture group and placebo group (with standard deviation 50% from mean value) the number of patients was calculated to be 25 per group. Expecting a drop-out rate of up to 15% in clinical trials, the number of patients per group was inflated to 30, in total 60 patients for the investigation. For sample size calculation, the online software (free access) was used [14].

### 2.8. Data Analysis

Normally distributed continuous data will be compared using the Student’s *t*-test and an analysis of variance, as appropriate. Skewed data will be compared using Kruskall–Wallis and Friedman test. Chi-square test will be used to analyze the incidence of gagging and rescue midazolam application and the success of patients’ blinding. Statistical analysis will be performed using the software Statistical Package for Social Science, version 22.0 (SPSS, IBM Corporation, New York, NY, USA).

## 3. Discussion

In this protocol, we describe a prospective randomized, patient, practitioner and assessor-blinded, single-center trial, which will compare real acupuncture with a placebo procedure in addition to current standard therapy for the treatment of gagging during transesophageal echocardiography (TEE). The aim of this pragmatic clinical investigation is to develop an effective, easily applied method to relieve the gag reflex using needling of acupoints CV24 and PC6 in patients scheduled for ambulatory TEE for cardiologic diagnostics.

Gagging during TEE is extremely unpleasant for patients and may disturb the TEE-examiner, who is trying to perform a safe and effective diagnostic procedure [15]. Premedication with midazolam was shown to improve patient comfort during and after TEE [16], however, extensive use of sedatives is associated with prolonged sedation, delayed recovery and the need for prolonged observation prior to safe discharge for ambulatory patients [17]. Acupuncture could be a safe and easily applied method to avoid pharmacological sedation in patients scheduled for an ambulatory TEE procedure.

In this project, we will use intradermal fixed needles for acupuncture during the TEE procedure for the first time. According to our experience, the practitioner, performing TEE, often comes into contact with long acupuncture needles placed at acupoint CV24, which is situated in the mentolabial groove (Figure 1). These accidental contacts may cause a painful reaction, which leads to the uncontrolled movement of patients during TEE examination. Thus, we have decided to use intradermal needles, which will not disturb the TEE examination (manipulation with TEE probe in the perioral area).

The combination of two acupoints, CV24 and PC6, which is used for the first time for this indication, will allow the relief of both gagging and nausea during TEE. From a practical point of view, the use of only two acupoints to relieve the gag reflex promises to be easier in clinical routine than the acupuncture of 10 various acupoints, which was reported in a previous trial for the treatment of stress and gagging in patients undergoing gastroscopy [18].

Although we have based our choice of control condition on experts’ recommendations for the optimal control procedures for clinical acupuncture trials [19], the use of a physiologically inactive placebo control condition may introduce at least two sorts of bias into the present investigation: (i) the inactive placebo condition lacks the unspecific physiological effect that might indirectly enhance the measured specific effect of acupuncture measured vs. inactive placebo only [20]; (ii) probably it is likely that some participants who will randomly receive the New Pyonex placebo needles will be able to distinguish them from real acupuncture, despite the use of tip pressure from the neural pen, which imitates the pricking sensation. Nevertheless, we will carefully monitor this important aspect during our investigation, interviewing the patients and practitioners about their opinion regarding the group allocation. 

In conclusion, we describe a proposal for a prospective randomized, patient, practitioner and assessor-blinded, single-center trial with two arms/groups: real acupuncture will be compared to placebo acupuncture as an addition to the current standard therapy.

### Trial Status

The participants are currently being recruited for the present study.

## Figures and Tables

**Table 1:** Interventions details by Standards for Reporting Interventions in Clinical Trials of Acupuncture (STRICTA) items.

Item	Detailed Items	Description
1. Acupuncture rationale	1a) Style of acupuncture 1b) Reasoning for treatment provided based on historical context, literature sources, and/or consensus methods, with references where appropriate 1c) Extent to which treatment was varied	Western medical acupuncture Acupuncture methods described as effective in previous pilot investigations (references [6,7,8,9,10,11]) Standardized acupuncture for each patient, no individual variation
2. Details of needling	2a) Number of needle insertions per subject per session (mean and range where relevant) 2b) Names (or location if no standard name) of points used (uni/bilateral) 2c) Depth of insertion, based on a specified unit of measurement or on a particular tissue level 2d) Response sought 2e) Needle stimulation 2f) Needle retention time 2 g) Needle type (diameter, length, and manufacturer or material)	Three intradermal needles *Chengjiang* (CV24) midline acupoint *Neiguan* (PC6) bilateral 5 mm at CV24; 1.5 mm at PC6 No response sought No needle stimulation Until the end of TEE (max. 30 min) Intradermal “Spinex” needle (0.14 mm × 6 mm, Seirin Corp. Japan) at CV24; indwelling ear acupuncture “New Pyonex” needle (0.2 mm × 1.5 mm, Seirin Corp. Japan) at PC6
3. Treatment regimen	3a) Number of treatment sessions 3b) Frequency and duration of treatment sessions	One session Once for each patient
4. Other components of treatment	4a) Details of other interventions administered to the acupuncture group 4b) Setting and context of treatment, including instructions to practitioners, and information and explanations to patients	None Each patient will be informed about acupuncture or placebo procedure against gagging during TEE ^1^
5. Practitioner background	5) Description of participating acupuncturists (qualification or professional affiliation, years in acupuncture practice, other relevant experience)	Licensed medical acupuncturist with more than 10 years of acupuncture practice
6. Control of comparator interventions	6a) Rationale for the control or comparator in the context of the research question, with sources that justify this choice 6b) Precise description of the control or comparison group. If sham acupuncture or any other type of acupuncture-like control is used, provide details as for items 1 to 3, above	To study the efficacy and safety of acupuncture as an additional therapy in the relief of gagging during a routine TEE procedure Placebo needles will be placed over the same sites in patients from the control group as in the acupuncture group

^1^ TEE: transesopageal echocardiography.

## Supplementary Materials

The following are available online at https://www.mdpi.com/2305-6320/7/4/17/s1, Figure S1: Schedule of enrollment, interventions and assessment.

## References

[1] Davies A.E., Kidd D., Stone S.P., MacMahon J. (1995). Pharyngeal sensation and gag reflex in healthy subjects. Lancet.

[2] Hughes T.A.T., Wiles C.M. (1996). Palatal and pharyngeal reflexes in health and motor neuron disease. J. Neurol. Neurosurg. Psychiatry.

[3] Bassi G.S., Humphris G.M., Longman L.P. (2004). The etiology and management of gagging: A review of the literature. J. Prosthet. Dent..

[4] Khongkaew J., Sahasthas D., Potat T., Thammawirat P. (2016). Modified mallampati classification in determining the success of unsedated transesophageal echocardiography procedure in patients with heart disease: Simple but efficient. Cardiovasc. Ultrasound..

[5] Prashanti E., Sumanth K.N., Renjith George P., Karanth L., Soe H.H. (2015). Management of gag reflex for patients undergoing dental treatment. Cochrane Database Syst. Rev..

[6] Fiske J., Dickinson C. (2001). The role of acupuncture in controlling the gagging reflex using a review of ten cases. Br. Dent. J..

[7] Rosted P., Bundgaard M., Fiske J., Pedersen A.M. (2006). The use of acupuncture in controlling the gag reflex in patients requiring an upper alginate impression: An audit. Br. Dent. J..

[8] Mitchell J., Jeffrey S., Lochhead V. (2008). Use of acupuncture to reduce gagging during insertion of an oral airway. Anaesthesia.

[9] Rösler A., Otto B., Schreiber-Dietrich D., Steinmetz H., Kessler K.R. (2003). Single-needle acupuncture alleviates gag reflex during transesophageal Echocardiography: A blinded, randomized, controlled pilot trial. J. Alternat. Complement. Med..

[10] Bilello G., Fregapane A. (2014). Gag reflex control through acupuncture: A case series. Acupunct. Med..

[11] Goel H., Mathur S., Sandhu M., Jhingan P., Sachdev V. (2017). Effect of Low-level LASER Therapy on P6 Acupoint to Control Gag Reflex in Children: A Clinical Trial. J. Acupunct. Meridian Stud..

[12] MacPherson H., White A., Cummings M., Jobst K., Rose K., Niemtzow R. (2002). STandards for Reporting Interventions in Controlled Trails of Acupuncture. Standards for reporting interventions in controlled trials of acupuncture: The STRICTA recommendations. STandards for Reporting Interventions in Controlled Trails of Acupuncture. Acupunct. Med..

[13] Usichenko T.I., Lysenyuk V.P., Groth M.H., Pavlovic D. (2003). Detection of ear acupuncture points by measuring the electrical skin resistance in patients before, during and after orthopedic surgery performed under general anesthesia. Acupunct. Electrother. Res..

[14] Inference for Means: Comparing Two Independent Samples. https://www.stat.ubc.ca/~rollin/stats/ssize/n2.html.

[15] Chan K.L., Cohen G.I., Sochowski R.A., Baird M.G. (1991). Complications of transesophageal echocardiography in ambulatory adult patients: Analysis of 1500 consecutive examinations. J. Am. Soc. Echocardiogr..

[16] Aeschbacher B.C., Portner M., Fluri M., Meier B., Lüscher T.F. (1998). Midazolam premedication improves tolerance of transesophageal echocardiography. Am. J. Cardiol..

[17] Maurice-Szamburski A., Auquier P., Viarre-Oreal V., Cuvillon P., Carles M., Ripart J., Honore S., Triglia T., Loundou A., Leone M. (2015). Effect of sedative premedication on patient experience after general anesthesia: A randomized clinical trial. JAMA.

[18] Cahn A.M., Carayon P., Hill C., Flammant R. (1978). Acupuncture in gastroscopy. Lancet.

[19] Lundeberg T., Lund I., Näslund J., Thomas M. (2008). The Emperors sham—Wrong assumption that sham needling is sham. Acupunct. Med..

[20] Vickers A.J. (2002). Placebo controls in randomized trials of acupuncture. Eval. Health Prof..

