# Supplementary material for: Acupuncture for Relief of Gag Reflex in Patients Undergoing Transoesophageal Echocardiography—A Protocol for a Randomized Placebo-Controlled Trial
Source: Medicines (Basel). 2020 Mar 31;7(4):17. doi: 10.3390/medicines7040017 (PMC7235877; doi:10.3390/medicines7040017)
Supplement: Supplementary file 1 [file medicines-07-00017-s001.pdf]

Supplementary figure**.** Schedule of enrolment, interventions, and assessments

	**STUDY PERIOD**					
	**Enrolment**	**Allocation**	**Post-allocation**			**Close-out**
**TIMEPOINT****	***T_0 - 10 min***	***T_0***	***T_0 + 5min***	***T_0 + 10 min***	***T_0 + 15 min***	***T_0 + 20 min***
**ENROLMENT:**						
Eligibility screen	X					
Informed consent	X					
Randomization		X				
Allocation		X				
**INTERVENTIONS:**						
Real acupuncture						
Placebo acupuncture						
**ASSESSMENTS:**						
Incidence and intensity of gagging						X
Incidence and intensity of nausea						X
Use of rescue medication						X
Patients' satisfaction with relief of unwanted side effects						X
Participants' opinion to group allocation						X
Heart rate and oxygen saturation						
Side effects of acupuncture						X
